# Palliative Care Coordination Interventions for Caregivers of Community-Dwelling Individuals with Dementia: An Integrative Review

**DOI:** 10.3390/nursrep14030130

**Published:** 2024-07-17

**Authors:** Diana Layne, Ayaba Logan, Kathleen Lindell

**Affiliations:** 1 College of Nursing, Medical University of South Carolina, Charleston, SC 29425, USA; lindellk@musc.edu; 2 Academic Affairs, Medical University of South Carolina, Charleston, SC 29425, USA; loganay@musc.edu

**Keywords:** patient care management, palliative care, systematic review, dementia and caregivers

## Abstract

Alzheimer’s disease is a serious illness with a protracted caregiving experience; however, care coordination interventions often lack the inclusion of palliative care. The purpose of this integrative review is to identify and synthesize existing care coordination interventions that include palliative care for individuals with dementia and their caregivers living in community settings. The Whittemore and Knafl framework guided the review, with data analysis guided by the SELFIE framework domains. Study quality was assessed using the Mixed Methods Appraisal Tool, while the Preferred Reporting Items for Systematic Reviews and Meta-Analyses (PRISMA) guidelines informed reporting results. Nine care coordination interventions involving family caregivers across eighteen publications were identified. Only a single intervention explicitly mentioned palliative care, while the remaining interventions included traditional palliative care components such as advance care planning, symptom management, and emotional support. Many of the identified interventions lacked theoretical grounding and were studied in non-representative, homogeneous samples. Further research is needed to understand the lived experiences of people with dementia and their caregivers to alleviate care coordination burden.

## 1. Introduction

The World Health Organization estimates more than 55 million people have dementia worldwide, with nearly 10 million new cases annually [1]. Alzheimer’s disease is the most common form of dementia and affects approximately 6.7 million Americans aged 65 and older or 1 in 9 people over the age of 65 [2]. Global estimates of persons with Alzheimer’s disease are approximately 22% of those aged 50 and above [3]. Future estimates suggest that by 2050, 152 million people globally will be living with Alzheimer’s disease and related dementias [4]. The often-extended illness trajectory for persons living with Alzheimer’s Disease and Related Dementias (ADRDs) coupled with progressive decreases in cognitive functioning creates a complex environment for symptom self-management, often requiring family caregiver assistance. A recent report from the Alzheimer’s Association indicates that 70% of dementia caregivers within the United States report that coordinating care is stressful [5] compared to 54% of dementia carers globally [6]. Furthermore, 50% of global dementia caregivers report that their health has suffered as a result of caregiving responsibilities, despite expressing positive sentiments regarding their caregiving role [6]. In 2023, more than 11 million Americans provided unpaid care to someone living with ADRDs [7]. Informal care hours for those living with dementia are estimated to be 133 billion hours annually [8]. According to the Centers for Disease Control, an informal caregiver provides regular care or assistance to a friend or family member who has a health problem or disability [9]. Informal caregivers commonly report challenges with care coordination activities. In the 2020 update of the Caregiving in the US report, 31% of caregivers reported having at least some difficulty in coordinating care among their care recipient’s healthcare providers, previously reported at 23% in 2015 [10]. The Agency for Healthcare Research and Quality (AHRQ) [11] defines care coordination as “deliberately organizing patient care activities and sharing information among all participants concerned with a patient’s care to achieve safer and more effective care with a primary goal of meeting patients’ needs and preferences in the delivery of high-quality, high-value healthcare”. Examples of care coordination tasks assumed by informal caregivers of persons with ADRDs include managing medical appointment scheduling/attendance often for multiple providers, attendance at community programs (such as support groups or respite care), transportation needs, and assistance with activities of daily living. Consequences of poor care coordination include increased caregiver stress and burden [12] as well as fragmented care from multiple providers that lacks continuity, resulting in suboptimal outcomes for those living with ADRDs and their caregivers [13].

Management of ADRDs is complex and those diagnosed with ADRDs report needing assistance obtaining accurate and helpful information, understanding diagnosis and disease trajectory [14], living with the symptoms of dementia, learning to do things differently, and establishing coping mechanisms [15]. In contrast, caregivers report stressors associated with personal care, housekeeping, medication administration, financial responsibilities, and difficulty maintaining professional and social connections [16]. Concerns for the safety of the person living with ADRDs were also reported by caregivers in four primary areas: physical harm, economic harm, emotional harm, and relational harm [17]. Care coordination is a potential strategy for aiding persons living with ADRDs and their caregivers in managing their complex needs.

Care coordination is aimed at organizing complex systems of care to meet the needs and preferences of patients during the delivery of high-quality, high-value care [11]. Knowledge sharing, care planning, and aligning resources with patient and family needs are a few strategies used to achieve effective care coordination. These activities can be based in acute care settings such as hospitals, outpatient settings such as primary care, or community settings such as the Alzheimer’s Association Dementia Care Coordination program. Evidence suggests that high continuity of care improves outcomes for persons living with ADRDs [18,19,20].

### Problem Identification

Despite the serious nature of Alzheimer’s disease, care coordination interventions often lack integration of palliative care. Palliative care is specialized care for individuals living with serious illnesses aimed at providing symptom relief and improving quality of life for patients and families [21]. In addition to aiding persons living with ADRDs and caregivers with symptom management (e.g., agitation, pain), palliative care incorporates a holistic approach and also focuses on other aspects such as psychological, social, and spiritual needs for caregivers and care recipients [22]. A common misconception is that palliative care should only be offered when an individual is nearing death; however, palliative care is available and should be offered to all patients living with serious illnesses at any point in their care, including at diagnosis [23,24,25,26]. Early access to palliative care increases the opportunity for persons living with ADRDs to collaborate with caregivers to plan accordingly for future care based on individual needs and preferences, which becomes increasingly difficult as cognition declines over the illness trajectory. Existing systematic reviews have examined psychosocial interventions for persons living with ADRDs [27,28,29], the significance of caregiver interventions on caregiver outcomes [30,31,32,33,34,35], and mHealth applications [36]. However, none to date have specifically examined the integration of palliative care into care coordination interventions for community-dwelling persons with ADRDs. Current estimates suggest that approximately 80% of individuals with ADRDs are community-dwelling [37]. Thus, understanding the existing scope of care coordination interventions for those living with ADRDs and their caregivers is a critical step to improving outcomes. The aim of this integrative review is to synthesize existing evidence on care coordination interventions that integrate palliative care for community-dwelling persons with ADRDs and their caregivers.

## 2. Materials and Methods

Whittemore and Knafl’s framework was selected to guide this integrative review [38] since the primary goal was to synthesize existing evidence. This framework has five stages: problem identification, literature search, data evaluation, data analysis, and presentation. Employing the Whittemore and Knafl framework allowed for the inclusion of diverse methodologies, thus the included studies provide a more comprehensive view of available research. In addition, for reporting purposes, the Preferred Reporting Items for Systematic Reviews and Meta-Analyses (PRISMA) guidelines were followed, which includes a flow chart.

### 2.1. Literature Search Strategy

Following consensus by all team members on a defined search strategy, a systematic literature search was conducted by a single author. The electronic databases included Scopus^®^, PubMed, ProQuest Healthcare Administration Database, and seven databases within the EBSCOHost platform, including Medline, CINAHL Complete, Academic Search Premier, APA PsychInfo, and Health Source (Nursing/Academic Edition, Consumer Health Complete, and Psychology and Behavioral Sciences Collection). The searches were conducted using advanced adaptive keyword searching in each database to consistently use the same or similar search terms based on the taxonomy of the database. The final search was conducted in May of 2023 to find all the palliative care interventions with caregivers using the following terms: (“care coordination” OR “case management”) AND (“family caregivers” OR “informal caregivers” OR relatives OR family) AND (“palliative care”) AND (intervention) AND (dementia). Following the electronic databases search, a hand-search of personal libraries, articles found incidentally (i.e., Google Scholar), and those obtained from the reference lists of relevant articles were also included. Once the electronic search and hand search were completed, all citations were uploaded to Covidence [39] to complete screening and evaluation. Detailed search strategy and completed PRISMA diagram are included within the Supplementary Materials.

### 2.2. Inclusion and Exclusion Criteria

The inclusion and exclusion criteria were developed collaboratively during team meetings based on the stated aim. Specific time restrictions for inclusion were intentionally not included to ensure a comprehensive review of available interventions. Quantitative, qualitative, and mixed methods studies were included if (1) a care coordination intervention was described, (2) the intervention included informal dementia caregivers, and (3) palliative care was incorporated within the intervention. Excluded articles included those that (1) described interventions focused on healthcare workers and (2) were aimed at hospital processes such as nurse case management within the hospital. Informal caregivers were defined as spouses, parents, relatives, or friends.

### 2.3. Data Evaluation

Covidence [39] was used to screen, extract, and assess all manuscripts. All documents were uploaded into Covidence, where title abstract screening was mainly conducted by one reviewer (DL), followed by a shared full review by two authors (DL and KL), with a third author (AL) handling conflicts. The Mixed Methods Appraisal Tool (MMAT) [40] was used to assess the quality of all relevant articles after full-text review. What data to be extracted from each article were discussed and agreed upon as a team and then added to Covidence using the Data Extraction Template. Finally, the MMAT was created in Covidence using the Quality Assessment Template. Both were performed by one author (DL). The MMAT contains five questions to assess study quality based on study design. Studies that met all five criteria were categorized as high quality. Studies that addressed four criteria were categorized as moderate quality and those meeting three or fewer criteria were classified as low quality. (See Supplementary Materials for the search strategy and procedures employed in each of the databases or platform searches). Upon completion of the data extraction and quality assessment in Covidence, results were exported to Excel for further analysis.

### 2.4. Data Analysis

The Sustainable Integrated Chronic Care Models for Multi-morbidity: Delivery, Financing, and Performance (SELFIE) framework for integrated care for multi-morbidity was used to organize the results of this review [41] (Table 1). This framework utilizes the World Health Organization’s (WHO’s) six health system building blocks as separate domains within the model [42] organized into micro, meso, and macro levels, with the individual with multi-morbidity and their environment centrally located within the model [41]. The WHO’s six building blocks of health, which each encompass a single domain of the model, include service delivery, leadership and governance, workforce, financing, technologies and medical products, and information and research. Within this model, the individual and their environment are viewed with a holistic lens incorporating health, well-being, capabilities, self-management, needs, and preferences. Environmental factors such as housing, social network, finances, availability of community services, physical surroundings, and accessible transportation are also considered from the individual’s perspective. Within each of the model domains, micro components focus on the individual level, meso components are aimed at the community level, and macro components are aimed at health policy, infrastructure, and financially sustainable models of care. Monitoring at each of these levels within each domain is also incorporated. Interventions were categorized using the SELFIE domains at each applicable level (micro, meso, macro). Table 1 provides a description of each domain and examples of micro-, meso-, and macro-level activities.

## 3. Results

The initial search yielded 2693 articles. Following duplicate removal, 1743 abstracts and titles were screened for inclusion, with 250 assessed using full-text review. Following full-text review, a total of 18 manuscripts met the inclusion criteria (see Supplementary Materials). Overall, nine unique care coordination interventions involving caregivers and integrating palliative care were identified across the 18 publications. The named interventions included Admiral Nursing [43,44,45], Dementia Care Coordination [46], UCLA Alzheimer’s and Dementia Care/D-Care [47,48,49], ACCESS [50,51], PEACE [52,53], the MIND [54], COACH [55], the Live@Home [56,57], and Partners in Dementia Care (PDC) [58,59,60]. Most interventions (seven) addressed micro- and/or meso-level characteristics on the SELFIE framework domains and levels, but few (two) addressed macro-level characteristics across all six domains of the SELFIE model. The sample size ranged from 35 to 2150, with mostly white participants over 60 years old.

Manuscripts included results from four randomized control trials [50,51,54,59], two qualitative studies [52,57], three mixed methods [44,45,46], two cohort studies [50,55], and one case report [43]. Additionally, three program descriptions [48,53,58] and two study protocols [49,56] were published. Three theoretical frameworks—the Stress Process Model [52], Chronic Care model [50,59,60], and the European Association of Palliative Care (EAPC) domains of optimal palliative care—were used for developing one intervention described across three articles [43,44,45]. However, the remaining intervention descriptions lacked information on which, if any, theoretical/conceptual models were used to guide the development. Results from one study were used to inform the development of the Dementia Care Coordination (DCC) Program theory [46].

Most interventions were studied within the United States (72%), while the remaining interventions were studied in the United Kingdom (17%) and Norway (11%). Table 2 contains the intervention description, study design, sample characteristics, study aims, and results of included interventions. Several interventions had more than one publication reporting results, some of which used various study designs. For example, the Palliative Excellence in Alzheimer’s Care Efforts (PEACE) program reported results from a qualitative study [52] as well as published a program description [53]. Table 3 contains theoretical underpinnings, SELFIE framework domains and levels, and study quality for all nine interventions. Most interventions addressed a minimum of four or more of the six domains within the SELFIE model and interventions primarily focused on micro- and meso-level interventions. Most studies were categorized as low quality based on the MMAT criteria. Study protocols and program descriptions were not assessed for study quality.

### 3.1. Service Delivery

All interventions addressed service delivery at the micro level. These interventions were person-centered, involved caregivers, and were delivered using various mechanisms (via telephone, in-person, or a combination of in-person and telephone). Each intervention embraced shared decision-making, which is a key tenet of micro-level interventions within the service delivery domain. Six interventions addressed service delivery at the meso level—palliative excellence in Alzheimer’s care efforts (PEACE) [52,53], Admiral nursing [43], dementia care coordination (DCC) [46], hospital-based dementia care and community-based care [49], dementia guideline-based care [50], and Partners in Dementia Care (PDC) [58]—due to the distinct integration of community resource referrals. There were no interventions that addressed market regulation policies to integrate care across organizations and sections, or service availability and access, which was required at the macro level of service delivery within the model.

### 3.2. Leadership and Governance

All included interventions addressed the leadership and governance domain at a micro level through shared decision-making. Admiral nursing was the only intervention that did not specifically describe an individualized care plan. Four interventions were aligned with the meso level [49,50,52,58] because of the distinct integration of community resource referrals. None of the interventions addressed policy and action plans related to chronic diseases and multi-morbidity or political commitment, which would have been classified as macro-level interventions within this domain.

### 3.3. Workforce

All interventions addressed the workforce at the micro and meso levels; however, none addressed macro-level components. Each intervention included a named coordinator and multidisciplinary team that are aligned within the micro level of this domain. Further, all interventions also provided informal caregiver support that aligns with a meso-level intervention. None of the interventions included workforce demography match and/or educational and workforce planning, which would have been required to align with the macro level of the framework.

### 3.4. Financing

Only a single intervention addressed the financing domain at the micro level, which included information related to coverage and reimbursement and out-of-pocket costs [59]. Bass [59] reported a cost estimate for program sustainability of 60 USD – 80 USD monthly per dyad (person living with Alzheimer’s Disease and their identified care partner). Furthermore, no interventions included incentives for collaboration, risk adjustment, shared savings, secured budgets, or a business case that encompasses the meso level within this domain. Finally, none of the included interventions addressed equity and access, stimulating investments in innovative care models or financial systems for health and social care, which are macro components within the financing domain.

### 3.5. Technologies and Medical Products

Seven interventions addressed technologies and medical products at the micro level across ten publications [43,44,45,46,47,49,50,51,54,56,57,58,60], with one intervention also addressing meso-level components across two publications [50,51]. Integration of electronic medical records, case-management-specific software, and/or patient portals was incorporated within Admiral nursing, Dementia Care Coordination (DCC), health system and community-based dementia care program, dementia guideline-based disease management, PEACE, MIND, and COACH—interventions that addressed micro-level components of the technologies and medical products domain. The LIVE intervention incorporated an evaluation of currently used assistive technologies with recommendations of other potentially useful assistive technologies for the individual living with dementia and their caregiver based on individualized needs that also address the micro level. Shared information systems within the interventions and/or interoperable systems were included within PDC and described as the PDC care coordination information system (CCIS), which functioned to share information across agencies and act as a centralized care plan for enrolled participants. Additionally, no interventions addressed policies fostering technological innovation. However, the LIVE@Home intervention did facilitate access to technologies and medical products not currently in use based on individual assessments of the needs of enrolled participants.

### 3.6. Information and Research

Five interventions were categorized as addressing information and research at the micro level across eight publications [46,50,51,54,58,59,60]. Interventions that included individual-level data and/or individual risk prediction were categorized as micro-level interventions. None of the interventions addressed data ownership and protection, innovative research (methods), or risk stratification that aligns with the meso level within this domain. Macro-level components of this domain, including privacy and data protection legislation, policies that stimulate research in integrated care and multimorbidity, and access to information were not addressed by any intervention.

### 3.7. Integration of Palliative Care

Examples of palliative care integration within each intervention included activities such as disease-specific education, symptom management, and/or emotional support. Interventions that incorporated disease-specific education included dementia guideline-based care, MIND, PDC, Admiral nursing, and DCC. Interventions that included content related to symptom management included Admiral nursing, DCC, and dementia guideline-based care. Finally, descriptions of the MIND intervention specifically mentioned the provision of emotional support to both patients and caregivers.

## 4. Discussion

The aim of this integrative review was to synthesize existing evidence of care coordination interventions that integrate palliative care for community-dwelling PLWD and caregivers. The identified interventions provide a variety of resources, including care management from diagnosis through bereavement. Delivery of the interventions differs per intervention, with delivery by healthcare professionals, including physicians, advanced practice nurses, nurses, social workers, and community health workers delivering intervention components. Findings from this integrative review suggest that identified interventions were person-focused, with some community engagement; however, interventions addressing health policy, infrastructure for care coordination involving caregivers, and integrating palliative care are lacking. Additionally, sustainable financial models are needed to support these types of interventions. These findings are consistent with a recent National Academies of Medicine, Science, and Engineering (NAMSE) report [61], which recommended the Centers for Medicare and Medicaid Services investigate the value of collaborative care models as a benefit through Medicare Advantage programs, alternative payment models, and fee-for-service beneficiaries.

In comparative literature, Sydney et al. [62] conducted a systematic review of continuity, coordination, and transition of care for patients with serious and advanced illnesses and found methodological issues in measurement tools for patients with advanced illnesses and moderate evidence that interventions targeting continuity, coordination, and transitions in patients with serious illness improved patient and caregiver satisfaction, but had low evidence of impact on outcomes [62]. In another study, Bajwah et al. [63] conducted a meta-analysis that assessed the effectiveness and cost-effectiveness of hospital-based specialty palliative care (HSPC) compared to usual care for adults with advanced illness and their unpaid caregivers/families. They reviewed 42 randomized control trials evaluating the impact of HSPC on outcomes for patients or their unpaid caregivers/families, or both and found very low- to low-quality evidence suggesting that, compared to usual care, HSPC may offer small benefits for several person-centered outcomes while also increasing the chances of patients dying in their preferred place (measured by home death). They found no evidence that HSPC causes serious harm; however, the evidence was insufficient to draw strong conclusions and they recommended that more research, particularly including patient-centered outcomes, is needed [63].

### Strengths and Limitations

Several limitations exist that should be discussed. Due to the broad scope and heterogeneous nature of the included sample, integrative reviews are prone to error [38]. Only studies published in English were included in this review. Furthermore, dissertations and non-peer-reviewed literature were also excluded. Additional limitations include the varied sample sizes of the included studies, limited racial and ethnic diversity across study participants, and limitations in longitudinal study designs. Finally, definitions of key terms such as ‘caregiver’, ‘palliative care’, and ‘care coordination’ varied across studies, which increases the potential for the omission of applicable studies. Despite these limitations, the use of the MMAT to evaluate study quality and the inclusion of a medical librarian as part of the team to develop a comprehensive search strategy strengthened this review and its findings.

## 5. Conclusions

Additional research is needed to better understand the lived experience of patients with serious illnesses, including persons with ADRDs and their caregivers. This review synthesized existing care coordination interventions that integrate palliative care for community-dwelling individuals with ADRDs and their caregivers and found that most existing care coordination interventions that incorporate palliative care are not grounded in theoretical and/or conceptual models. Further, these interventions have been primarily tested in a homogenous population, which suggests that further studies using a more representative sample are necessary to ensure equitable access to comprehensive care coordination that integrates palliative care and caregivers.

## Figures and Tables

**Table 1:** SELFIE framework domains and levels [41].

Domain	Micro Level Characteristics	Meso Level Characteristics	Macro Level Characteristics
Service delivery	Person-centered, tailored, self-management, proactive, informal caregiver involvement, treatment interaction, continuity	Organizational and structural integration, continuous quality improvement system	Market regulation, policies to integrate care across organizations and sectors, service availability and access
Leadership and governance	Shared decision-making, individualized care planning, and coordination tailored to complexity	Supportive leadership, clear accountability, performance-based management, culture of shared vision, ambition, values	Policy and action plans on chronic diseases and multi-morbidity, political commitment
Workforce	Multi-disciplinary team, named coordinator, core group	Continuous (professional) development, informal caregiver support, new professional roles	Workforce-demography match, educational and workforce planning
Financing	Coverage and reimbursement, out-of-pocket costs, financial incentives	Incentives to collaborate, risk adjustment, shared savings, secured budget, business case	Financial system for health and social care, stimulating investments in innovative care models, equity and access
Technologies and medical products	EMRs and patient portals, E-health tools, assistive technologies, remote monitoring	Shared information systems, interoperable systems	Policies fostering technological innovation, access to technologies and medical products
Information and research	Individual level data, individual risk prediction	Data ownership and protection, innovative research (methods), risk stratification	Privacy and data protection management, policies that stimulate research in integrated care and multi-morbidity, access to information

**Table 2:** Intervention descriptions, study designs, sample characteristics, and results (n = 18).

Intervention Description (Country)	Author (Year)	Study Design	Sample Characteristics Caregivers (CG) PLWD	Purpose	Results
Admiral Nursing (United Kingdom)—Admiral nurses are dementia care specialists who support families affected by dementia from diagnosis through death. They provide bereavement support for family members following the death of the individual living with dementia.	Dening, 2018 [43]	Case report	Not applicable	Describe the Admiral Nurse case management program within the context of the European Association of Palliative Care recommendations for palliative care in dementia.	Not applicable; case report
	Piercy, 2018 [44]	Mixed methods	Not reported; service evaluation	Examine how well the integrated service provided support to those living with dementia and their carers and to understand their unique benefits, opportunities, and challenges.	Approximately 37.8% of the eligible population registered for service over 14 months, with most being self-referrals. Carers reported high satisfaction. Fully integrated service with the Admiral nurse and dementia advisor offering a novel approach to care coordination.
	Gridley, 2021 [45]	Mixed methods	35 dementia caregivers CG Mean Age—NR CG Race—NR CG Gender—54% female CG Relationship—63% spouse	Examine the experiences of caregivers who received versus did not receive Admiral nursing services.	Carers reported feeling ‘supported’ when receiving ongoing Admiral nursing services or professional services from someone with dementia care expertise who could maintain a meaningful relationship.
Dementia Care Coordination (DCC) (United States)—DCC provides in-depth personalized services, including disease-specific education, symptom management advice, advance care planning guidance, emotional support, and referrals to community support resources.	Nadash, 2019 [46]	Mixed methods	14 key information interviews with selected staff Demographics not reported 15 physicians surveyed Demographics not reported 136 caregivers	Identify key care model components for a Dementia Care Coordination (DCC) program and determine if model components are functioning as planned.	The DCC program was deemed successful in supporting caregivers and medical professionals in managing ADRDs. However, early access to resources was problematic due to timely identification of potential participants. Additionally, incompatible data systems among agencies made information sharing challenging.
Dementia care study (D-CARE) (United States)—Based on the UCLA Alzheimer’s and Dementia Care intervention and includes eighteen months of health system-based dementia care delivered by a nurse practitioner or physician (arm 1), community-based dementia care delivered by a dementia specialist certified as a care consultant (arm 2), or usual care with referral to Alzheimer’s Association as needed (arm 3).	Jennings, 2019 [47]	Observational retrospective cohort	322 decedents from the ADC program. CR Mean Age—86.7 CR Race—72% white, non-Hispanic CR Gender—54% male CG Relationship— 49% child	Describe end-of-life preferences and acute care and hospice utilization within the last 6 months of life for enrolled participants.	Majority of decedents (99%) had documented goals of care conversations, with greater than half having advanced care preferences documented (64%). Of those with documented preferences, the majority had a documented ‘do not resuscitate’ order (88%). Additionally, 89% of participants had documented desires to not have artificial nutrition, including withholding tube feeding. Finally, more than half of the cohort had no documented hospitalizations or ED visits within the last 6 months of life.
	Reuben, 2013 [48]	Program description	First 150 enrollees CR Mean Age—82.7 CR Race—76% white CR Gender—42% male CG Relationship—48% spouse	Describe preliminary findings for the first 150 enrollees in the ADC program.	Majority of participants were referred to a community-based organization for services (55%). Most participants were referred to support groups (73%) and/or the Alzheimer’s Association Safe Return Program (73%).
	Reuben, 2020 [49]	Study protocol	2150 English- or Spanish-speaking PWD and their family/friend caregivers	Compare cost-effectiveness and participant healthcare utilization between a health system and community-based dementia care program and usual care.	Not applicable; study protocol
Alzheimer’s disease Coordinated Care for San Diego Seniors (ACCESS) (United States)—Formally trained dementia care managers (primarily social workers) conducted a home assessment using a case management software system. The case management software system suggested guides for care planning based on individualized assessment results. Care managers also provided education on problem-solving skills and referrals to community services, and performed care coordination activities. Home reassessments were conducted biannually.	Vickrey, 2006 [50]	RCT	408 caregivers and persons with dementia dyads CG Mean Age—65.6 CG Race—87% white CG Gender—68.9% female CG Relationship—54.8% spouse PLWD Mean Age—80.1 PLWD Race—86.4% white PLWD Gender—54.9% female	Evaluate the effectiveness of a dementia guideline-based disease management program (Alzheimer’s disease Coordinated Care for San Diego Seniors (ACCESS)) on adherence to 23 dementia care guidelines and community service utilization of caregivers of persons with dementia.	Results indicate that guideline adherence per participant at follow-up was 63.9% for those who received the intervention, which was significantly higher than the control group at 32.9%. Additionally, intervention participants received higher quality care on 21 of 23 guidelines and higher community agency assistance compared to those receiving usual care.
ACCESS adaptation (United States)—Social workers delivered a common care management protocol based on the ACCESS study over 12 months, including an initial assessment to identify any issues (e.g., educational needs, establishing advance care planning, etc.) and reassessment at 6 months. Mode and intensity of intervention delivery included an in-person community-based approach with telephone follow-up compared to a strictly telephone delivery.	Chodosh et al., 2015 [51]	RCT	151 caregivers and persons with dementia dyads CG Mean Age—49.5 CG Race—77.7% Hispanic or Latino CG Gender—65.3% female CG Relationship—53.5% child PLWD Mean Age—73.1 PLWD Race—73.6% Hispanic or Latino PLWD Gender—62.5% female	Compare the cost and effectiveness of telephone-only versus in-person and telephone delivery of a care coordination program.	Care quality improved over time in both groups; however, no differences were noted in caregiver burden, care recipient problem behaviors, retention, and utilization across arms. The in-person delivery costs exceeded the telephone-only program.
Palliative Excellence in Alzheimer’s Care Efforts (PEACE) (United States) —integration of palliative care into ongoing primary care for persons with dementia. Primary focus areas of the program include advance care planning, symptom management, education on disease process, caregiver support, optimal utilization of community resources, and improved care coordination. In the later stages of dementia, all individuals are offered hospice care. Two clinical nurse specialists interview participants at enrollment and biannually to uncover and address any unmet needs.	Diwan, 2004 [52]	Qualitative	150 community-dwelling, predominately African American patient/caregiver dyads CG Mean Age—61.9 CG Race—79% African American CG Gender—77% female CG Relationship—50% daughters PLWD Mean Age—82 PLWD Race—82% African American PLWD Gender—75% female	Identify predictors of types of caregiver strain of community-dwelling patients with dementia who participated in the Palliative Excellence in Alzheimer’s Care Efforts (PEACE) program.	Three types of caregiver strain were identified, including role, personal, and emotion related to caregiving. Patient problem behaviors predicted all types of caregiver strain. Perceived lack of support from the healthcare team predicted personal and emotional strain, while higher income predicted role strain. Personal and role strain were also predicted by patient functional limitations.
	Shega et al., 2003 [53]	Program description	Not applicable	Describe the PEACE program.	Not applicable; program description
Maximizing Independence at home (MIND) (United States)—Interdisciplinary care coordination (community health worker, registered nurse, geriatric psychiatrist) including individualized care plan development based on identified needs, priorities and preferences, coordination, and referral to existing community services and care monitoring.	Tanner, 2015 [54]	RCT	303 elders with memory disorders and 278 caregivers PLWD demographics not reported CG Mean Age—67 CG Race—71% white CG Gender—75% female CG Relationship—47% child	Examine the MIND intervention impact on caregiver outcomes (caregiver unmet needs, burden, depression, quality of life).	No statistically significant difference in caregiver outcomes between groups. However, a clinically relevant improvement in the amount of caregiver time spent with the care recipient was observed.
Caring for the Older Adults and Caregiver at Home (COACH) (United States)—The COACH care coordination program provides hands-on care via telephone and home visits, with support from an interdisciplinary team (social worker, RN, geriatrician, geriatric psychiatrist, and geriatric pharmacist). Visits occur 1, 3, and 6 months after enrollment and more frequently, if necessary, with follow-up telephone visits every three months thereafter.	D’Souza, 2015 [55]	Cohort study	133 veterans PLWD Mean Age—82.5 PLWD Race—63.6% white PLWD Gender—98.5% male	Describe the COACH program, assess quality outcomes, and report participant characteristics and placement rates of the PLWD outside of the home within 12 months	No statistically significant difference in time to permanent placement in long-term care between intervention and referred groups. However, the authors note the small sample size as a limitation of the generalizability of findings. Caregiver satisfaction with intervention was highly rated, at 96%
The Learning, Innovation, Volunteer support, and Empowerment at Home Pathway (LIVE@Home) (Norway)—The LIVE@Home intervention includes a municipal coordinator offering routine contact aiding participants with learning about dementia, accessing necessary resources to facilitate living at home, volunteer support to reduce social deprivation and end of life care, and advance care planning assistance.	Huesbo et al., 2020 [56]	Study protocol	315 dyads	Describe the development, feasibility testing, and implementation process of the LIVE@Home.Path trial.	Not applicable; study protocol
	Faeo, 2020 [57]	Qualitative	16 dyads (person living with dementia and caregiver), 2 care coordinators with their leader, Summarized demographics not reported	Understand the coordinator role within the LIVE@Home program and how a coordinator may empower those people living with dementia with decision-making processes.	Three themes emerged as primary functions of the coordinator: being a safety net, being a pathfinder, and being a source of emotional care and support. Coordinators identified having a trusting leader and working environment as critical to their success. Coordinators also found it challenging to build trusting relationships with the person living with dementia to empower decision-making.
Partners in Dementia Care (PDC) (United States)—PDC has three components: initial assessment, action plan, and ongoing monitoring and reassessment.	Judge, 2011 [58]	Program description	93 veterans with dementia and 90 family caregivers CG Mean Age—69.2 CG Race—84.4% white CG Gender—92.2% female CG Relationship—67% spouse PLWD Mean Age—80 PLWD Race—84.9% white PLWD Gender—94.6% male	Provide a detailed description of Partners in Dementia Care: A care coordination intervention for veterans with dementia and their family caregivers.	Not applicable; program description
	Bass, 2013 [59]	RCT	486 caregivers and 508 veterans CG Mean Age—69.1 CG Race—81% white CG Gender—94.9% female CG Relationship—NR Veteran Mean Age—NR Veteran Race—NR Veteran Gender—97.5% male	Examined the effectiveness of the Partners in Dementia Care (PDC) in a larger, more representative population	Caregivers demonstrated significant improvements in unmet needs, three types of caregiver strain (role captivity, physical health strain), depression, and use of support resources.
	Morgan, 2019 [60]	RCT	294 caregivers and veterans (Control/Intervention) CG Mean Age—(79.8/79.1) CG Race—(94% white/81% white) CG Gender—NR CG Relationship—(86% spouse/72% spouse) Veteran Mean Age—(72.1/68.9) Veteran Race—NR Veteran Gender—NR	Evaluate whether Partners in Dementia care influences VHA and non-VHA inpatient and ED use by veterans with dementia.	For veterans who lived closer to VA medical centers, participants in PDC demonstrated an increase over time (*p* < 0.01) in the likelihood of seeking VA care over non-VA care.

**Table 3:** Intervention, theoretical underpinning, SELFIE framework domains and Levels, and study quality (n = 18).

Author (Year)	Intervention	Theoretical Underpinnings	SELFIE Framework Domains and Levels	Study Quality
Dening, 2018 [43]	Admiral Nursing	EAPC domains of optimal palliative care in older people with dementia	Service delivery—Micro, Meso; Leadership and governance—Micro; Workforce—Micro, Meso; Technologies and medical products—Micro	Not applicable
Piercy, 2018 [44]				Low
Gridley, 2021 [45]				High
Nadash, 2019 [46]	Dementia Care Coordination (DCC)	None reported however DCC Program Theory following data analysis	Service delivery-Micro, Meso Leadership & governance-Micro Workforce-Micro, Meso, Macro Technologies & medical products-Micro Information & research-Micro	Low
Jennings, 2019 [47]	The UCLA Alzheimer’s and Dementia Care (ADC)	None reported	Service delivery—Micro, Meso Leadership and governance—Micro, Meso	Not applicable
Reuben, 2013 [48]				Moderate
Reuben, 2020 [49]	Dementia Care Study (D-Care)	None reported	Workforce—Micro, Meso Technologies and medical products—Micro	Not applicable
Vickrey, 2006 [50]	Alzheimer’s disease Coordinated Care for San Diego Seniors (ACCESS)	Chronic Care Model	Service delivery—Micro, Meso Leadership and governance—Micro, Meso Workforce—Micro, Meso Technologies and medical products—Micro, Meso Information and research—Micro	Moderate
Chodosh et al., 2015 [51]				High
Diwan, 2004 [52]	Palliative Excellence in Alzheimer’s Care Efforts (PEACE)	Stress Process Model	Service delivery—Micro, Meso Leadership and governance—Micro, Meso Workforce—Micro, Meso	Not applicable
Shega et al., 2003 [53]				Low
Tanner, 2015 [54]	Maximizing Independence at home (MIND)	None reported	Leadership and governance—Micro Workforce—Micro, Meso Technologies and medical products—Micro Information and research—Micro Service delivery—Micro	Low
D’Souza, 2015 [55]	Caring for Older Adults and Caregiver at Home (COACH)	None reported	Service delivery—Micro Leadership and governance—Micro Workforce—Micro, Meso Information and research—Micro	Low
Huesbo et al., 2020 [56]	Learning, Innovation, Volunteer support, and Empowerment at Home (LIVE@Home)	None reported	Service delivery—Micro Leadership and governance—Micro Workforce—Micro, Meso Technologies and medical products—Micro	High
Faeo, 2020 [57]				Not applicable
Judge, 2011 [58]	Partners in Dementia Care (PDC)	Chronic Care Model	Service delivery—Micro, Meso, Macro Leadership and governance—Micro, Meso Workforce—Micro, Meso Financing—Micro Technologies and medical products—Micro, Meso Information and research—Micro	Moderate
Bass, 2013 [59]				Not applicable
Morgan, 2019 [60]				Low

## Data Availability

The raw data supporting the conclusions of this article will be made available by the authors on request.

## Supplementary Materials

The following supporting information can be downloaded at: https://www.mdpi.com/article/10.3390/nursrep14030130/s1, File S1: Detailed search strategy and completed PRISMA diagram.

## Public Involvement Statement

No public involvement in any aspect of this research.

## Guidelines and Standards Statement

This manuscript was drafted against the PRISMA 2020 guidelines for systematic reviews [64].
