# Supplementary material for: Palliative Care Coordination Interventions for Caregivers of Community-Dwelling Individuals with Dementia: An Integrative Review
Source: Nurs Rep. 2024 Jul 17;14(3):1750–68. doi: 10.3390/nursrep14030130 (PMC11270266; doi:10.3390/nursrep14030130)
Supplement: Supplementary file 1 [file nursrep-14-00130-s001.zip › nursrep-3014855-supplementary.pdf]

Care Coordination Interventions with Caregivers 2

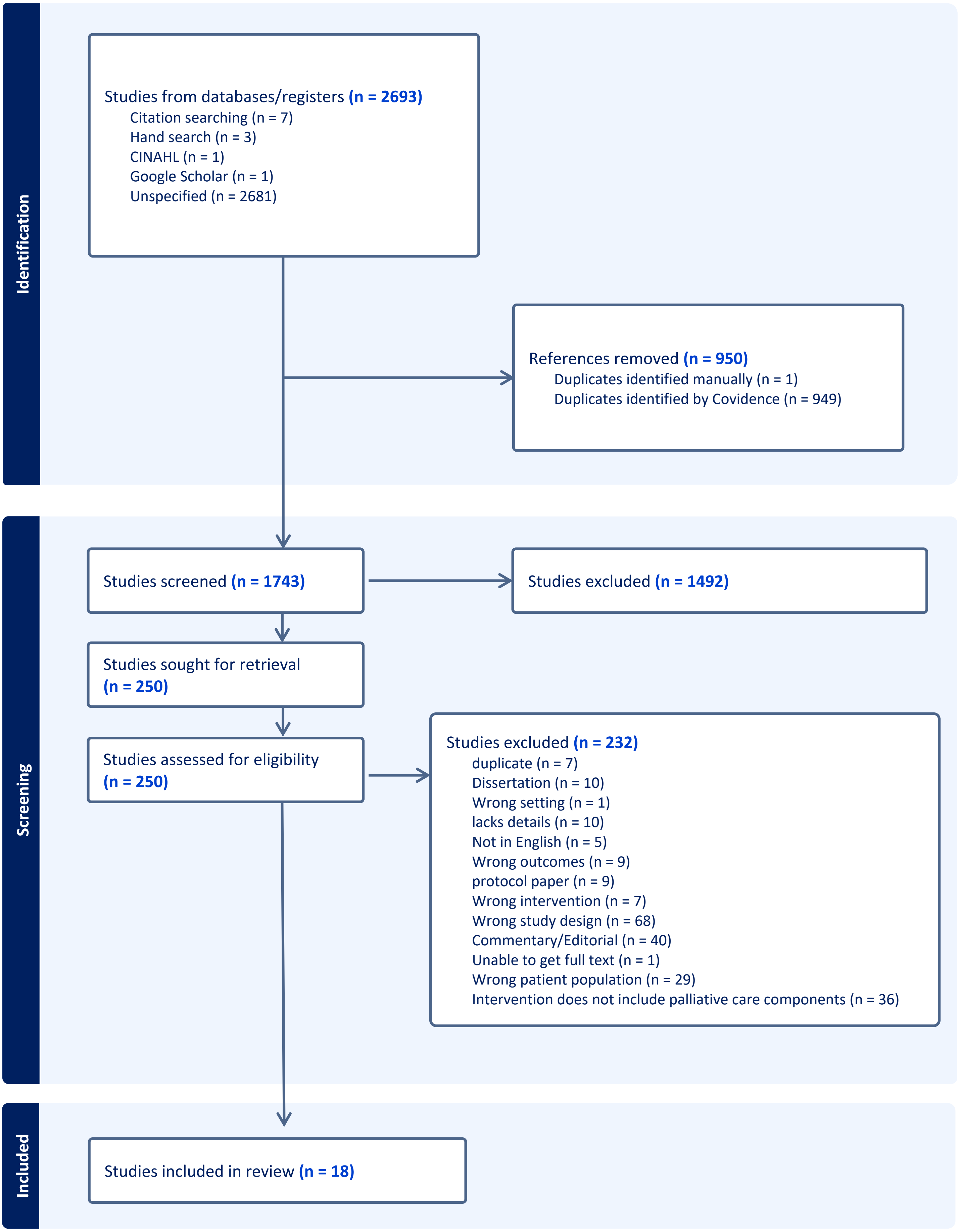

6th July 2023

covidence

I. EBSCOHost Final Search

(("care coordination" or "case management") AND (family caregivers or informal caregivers or relatives or family)) AND (palliative care) AND intervention) AND dementia.

5 documents

https://search-ebscohost-com.ezproxy-v.musc.edu/login.aspx?direct=true&AuthType=shib&db=ccm&db=aph&db=agr&db=awh&db=nsm&db=asf&db=buh&db=cph&db=i3h&db=nlebk&db=eft&db=eric&db=hev&db=zbh&db=funk&db=8gh&db=hxh&db=hch&db=khh&db=lii&db=lxh&db=ulh&db=f5h&db=cmedm&db=mih&db=mth&db=n5h&db=prh&db=tfh&db=pdh&db=pbh&db=psyh&db=bwh&db=rlh&db=sch&db=trh&db=tth&db=voh&db=lfh&db=b9h&db=nmr&db=e872sww&db=pwh&db=e865sww&db=e000xna&db=c9h&db=nlabk&db=e866sww&db=e871sww&db=e867sww&db=e869sww&db=e864sww&db=e870sww&bquery=(+(+%26quot%3bcare+coordination%26quot%3b+or+%26quot%3bcase+management%26quot%3b+)+AND+(+family+caregivers+or+informal+caregivers+or+relatives+or+family+)+)+AND+(+palliative+care)+AND+intervention+)+AND+dementia&dli0=NL&dlv0=Y&dld0=nlabk&type=1&searchMode=Standard&site=ehost-live&custid=s9006920

II. Scopus

TITLE-ABS-KEY (("care coordination" OR "case management") AND ("family caregivers" OR "informal caregivers" OR relatives OR family) AND ("palliative care" OR intervention) AND dementia)

75 results

III. ProQuest Healthcare Administration

https://www-proquest-com.ezproxy-v.musc.edu/search/1956142?accountid=36330

Select all	Set	Search	Databases	Results	Actions
Select item 3	**S3**	(("care coordination" OR "case management") AND ("family caregivers" OR "informal caregivers" OR relatives OR family) AND ("palliative care" OR intervention) AND dementia)Database: Healthcare Administration Database	Healthcare Administration Database	**1,503**	Actions

covidence

Select all	Set	Search	Databases	Results	Actions
Select item 2	**S2**	(("care coordination" OR "case management") AND ("family caregivers" OR "informal caregivers" OR relatives OR family) AND ("palliative care" OR intervention) AND dementia)Database: Healthcare Administration Database	Healthcare Administration Database	**1,503**	Actions
Select item 1	**S1**	(("care coordination" or "case management") AND (family caregivers or informal caregivers or relatives or family)) AND ("palliative care" OR intervention) AND dementia Database: Healthcare Administration Database	Healthcare Administration Database	**1,506**	Actions

EBSCOhost Research Databases Search Screen - Advanced Search Database :

CINAHL Complete;Academic Search Premier;Agricola;Alt HealthWatch;Newswires;Applied Science & Technology Full Text (H.W. Wilson);Business Source Premier;Computer Source;Criminal Justice Abstracts with Full Text;eBook Collection (EBSCOhost);Education Full Text (H.W. Wilson);ERIC;European Views of the Americas: 1493 to 1750;Fuente Académica;Funk & Wagnalls New World Encyclopedia;GreenFILE;Health Source - Consumer Edition;Health Source: Nursing/Academic Edition;History Reference Center;Library Literature & Information Science Index (H.W. Wilson);Library, Information Science & Technology Abstracts;MAS Ultra - School Edition;MasterFILE Premier;MEDLINE;Middle Search Plus;Military & Government Collection;Newspaper Source Plus;Primary Search;Professional Development Collection;APA PsycArticles;Psychology and Behavioral Sciences Collection;APA PsycInfo;Regional Business News;Religion and Philosophy Collection;Science Reference Center;Teacher Reference Center;TOPICsearch;Vocational and Career Collection;Literary Reference Center;Small Business Reference Center;Web News;AHFS Consumer Medication Information;Consumer Health Reference eBook Collection;Points of View Reference Center;MAS Reference eBook Collection;eBook Academic Collection (EBSCOhost);Consumer Health Complete - EBSCOhost;Audiobook Collection (EBSCOhost);Middle Search Reference eBook Collection;History Reference eBook Collection;Primary Search Reference eBook Collection;Science Reference eBook Collection;MasterFILE Reference eBook Collection;Literary Reference eBook Collection

Databases By Document Count

	Name	Hit Count
	All Databases	
1	MEDLINE	102
2	CINAHL Complete	74
3	Academic Search Premier	69
4	APA PsycInfo	54
5	Health Source: Nursing/Academic Edition	27
6	Consumer Health Complete - EBSCOhost	20

	Name	Hit Count
7	Psychology and Behavioral Sciences Collection	16
~~8~~	~~eBook Collection (EBSCOhost)~~	~~3~~
~~9~~	~~eBook Academic Collection (EBSCOhost)~~	~~3~~
10	Education Full Text (H.W. Wilson)	2
11	ERIC	2
12	Masterfile Premier	2
13	Newspaper Source Plus	2
14	APA PsycArticles	2
15	Criminal Justice Abstracts with Full Text	1
16	Religion and Philosophy Collection	1
17	Science Reference Center	1

Search Terms for EBSCOHost :

(("care coordination" or "case management") AND (family caregivers or informal caregivers or relatives or family)) AND (palliative care OR intervention) AND dementia

321 Results Before Duplicates removed automatically

136 Dups automatically removed

https://ezproxy.musc.edu/login?url=https://search.ebscohost.com/login.aspx?direct=true&db=ccm&db=aph&db=agr&db=awh&db=nsm&db=asf&db=buh&db=cph&db=i3h&db=nlebk&db=eft&db=eric&db=hev&db=zbh&db=funk&db=8gh&db=hxh&db=hch&db=khh&db=lii&db=lxh&db=ulh&db=f5h&db=cmedm&db=mih&db=mth&db=n5h&db=prh&db=tfh&db=pdh&db=pbh&db=psyh&db=bwh&db=rlh&db=sch&db=trh&db=tth&db=voh&db=lfh&db=b9h&db=nmr&db=l0h&db=e872sww&db=pwh&db=e865sww&db=e000xna&db=c9h&db=nlabk&db=e866sww&db=e871sww&db=e867sww&db=e869sww&db=e864sww&db=e870sww&bquery=(+(+%26quot%3bcare+coordination%26quot%3b+or+%26quot%3bcase+management%26quot%3b+)+AND+(+family+caregivers+or+informal+caregivers+or+relatives+or+family+)+)+AND+(+palliative+care+OR+intervention+)+AND+dementia&dli0=NL&dlv0=Y&dld0=nlabk&type=1&searchMode=Standard&site=ehost-live

IV. PubMed

Search: **("care coordination" OR "case management") AND ("family caregivers" OR "informal caregivers" OR relatives OR family) AND ((intervention) OR ("palliative care")) AND (dementia)**<br><br>Sort by: **Most Recent**("care coordination"[All Fields] OR "case management"[All Fields]) AND ("family caregivers"[All Fields] OR "informal caregivers"[All Fields] OR ("family"[MeSH Terms] OR "family"[All Fields] OR "relative"[All Fields] OR "relatives"[All Fields] OR "relative s"[All Fields] OR "relatively"[All Fields]) OR ("familialities"[All Fields] OR "familiality"[All Fields] OR "familially"[All Fields] OR "familials"[All Fields] OR "familie"[All Fields] OR "family"[MeSH Terms] OR "family"[All Fields] OR "familial"[All Fields] OR "families"[All Fields] OR "family s"[All Fields] OR "familys"[All Fields])) AND ("intervention s"[All Fields] OR "interventions"[All Fields] OR "interventive"[All Fields] OR "methods"[MeSH Terms] OR "methods"[All Fields] OR "intervention"[All Fields] OR "interventional"[All Fields] OR "palliative care"[All Fields]) AND ("dementia"[MeSH Terms] OR "dementia"[All Fields] OR "dementias"[All Fields] OR "dementia s"[All Fields]) **Translations relatives:** "family"[MeSH Terms] OR "family"[All Fields] OR "relative"[All Fields] OR "relatives"[All Fields] OR "relative's"[All Fields] OR "relatively"[All Fields] **family:** "familialities"[All Fields] OR "familiality"[All Fields] OR "familially"[All Fields] OR "familials"[All Fields] OR "familie"[All Fields] OR "family"[MeSH Terms] OR "family"[All Fields] OR "familial"[All Fields] OR "families"[All Fields] OR "family's"[All Fields] OR "familys"[All Fields] **intervention:** "intervention's"[All Fields] OR "interventions"[All Fields] OR "interventive"[All Fields] OR "methods"[MeSH Terms] OR "methods"[All Fields] OR "intervention"[All Fields] OR "interventional"[All Fields]**dementia:** "dementia"[MeSH Terms] OR "dementia"[All Fields] OR "dementias"[All Fields] OR "dementia's"[All Fields]	114	14:16:57

PubMed

**Search: ("care coordination" OR "case management") AND ("family caregivers" OR "informal caregivers" OR relatives or family) AND (intervention)**<br><br>("care coordination"[All Fields] OR "case management"[All Fields]) AND ("family caregivers"[All Fields] OR "informal caregivers"[All Fields] OR ("family"[MeSH Terms] OR "family"[All Fields] OR "relative"[All Fields] OR "relatives"[All Fields] OR "relative s"[All Fields] OR "relatively"[All Fields]) OR ("familialities"[All Fields] OR "familiality"[All Fields] OR "familially"[All Fields] OR "familials"[All Fields] OR "familie"[All Fields] OR "family"[MeSH Terms] OR "family"[All Fields] OR "familial"[All Fields] OR "families"[All Fields] OR "family s"[All Fields] OR "familys"[All Fields])) AND ("intervention s"[All Fields] OR "interventions"[All Fields] OR "interventive"[All Fields] OR "methods"[MeSH Terms] OR "methods"[All Fields] OR "intervention"[All Fields] OR "interventional"[All Fields])	2,691	15:34:11

**Translations relatives:** "family"[MeSH Terms] OR "family"[All Fields] OR "relative"[All Fields] OR "relatives"[All Fields] OR "relative's"[All Fields] OR "relatively"[All Fields] **family:** "familialities"[All Fields] OR "familiality"[All Fields] OR "familially"[All Fields] OR "familials"[All Fields] OR "familie"[All Fields] OR "family"[MeSH Terms] OR "family"[All Fields] OR "familial"[All Fields] OR "families"[All Fields] OR "family's"[All Fields] OR "familys"[All Fields] **intervention:** "intervention's"[All Fields] OR "interventions"[All Fields] OR "interventive"[All Fields] OR "methods"[MeSH Terms] OR "methods"[All Fields] OR "intervention"[All Fields] OR "interventional"[All Fields]		
